# Primary Amine Oxidase of *Escherichia coli* Is a Metabolic Enzyme that Can Use a Human Leukocyte Molecule as a Substrate

**DOI:** 10.1371/journal.pone.0142367

**Published:** 2015-11-10

**Authors:** Heli Elovaara, Teija Huusko, Mikael Maksimow, Kati Elima, Gennady G. Yegutkin, Mikael Skurnik, Ulrich Dobrindt, Anja Siitonen, Michael J. McPherson, Marko Salmi, Sirpa Jalkanen

**Affiliations:** 1 MediCity Research Laboratory, University of Turku, Turku, Finland; 2 Department of Medical Microbiology and Immunology, University of Turku, Turku, Finland; 3 Turku Graduate School of Biomedical Sciences, Turku, Finland; 4 Department of Medical Biochemistry and Genetics, University of Turku, Turku, Finland; 5 Department of Bacteriology and Immunology, Medicum, Immunobiology, Research Programs Unit, University of Helsinki, Helsinki, Finland and Helsinki University Central Hospital Laboratory Diagnostics, Helsinki, Finland; 6 Institute of Hygiene, University of Münster, Münster, Germany; 7 Bacteriology Unit, Department for Infectious Disease Surveillance and Control, National Institute for Health and Welfare, Helsinki, Finland; 8 Astbury Centre for Structural Molecular Biology, Institute of Molecular and Cellular Biology, Faculty of Biological Sciences, University of Leeds, Leeds, United Kingdom; National Research Council of Italy (CNR), ITALY

## Abstract

*Escherichia coli* amine oxidase (ECAO), encoded by the *tynA* gene, catalyzes the oxidative deamination of aromatic amines into aldehydes through a well-established mechanism, but its exact biological role is unknown. We investigated the role of ECAO by screening environmental and human isolates for *tynA* and characterizing a *tynA*-deletion strain using microarray analysis and biochemical studies. The presence of *tynA* did not correlate with pathogenicity. In *tynA+ Escherichia coli* strains, ECAO enabled bacterial growth in phenylethylamine, and the resultant H_2_O_2_ was released into the growth medium. Some aminoglycoside antibiotics inhibited the enzymatic activity of ECAO, which could affect the growth of *tynA+* bacteria. Our results suggest that *tynA* is a reserve gene used under stringent environmental conditions in which ECAO may, due to its production of H_2_O_2_, provide a growth advantage over other bacteria that are unable to manage high levels of this oxidant. In addition, ECAO, which resembles the human homolog hAOC3, is able to process an unknown substrate on human leukocytes.

## Introduction

Primary amine oxidases (EC 1.4.3.21, PrAOs, also known as copper-containing amine oxidases, CAOs) are expressed in mammals, plants, and bacteria. These enzymes catalyze the oxidation of primary amines into aldehyde through the reaction R-CH_2_-NH_2_ + O_2_ + H_2_O → R-CHO + H_2_O_2_ + NH_3_. The preferred amine substrate depends on the specific amine oxidase, and the aldehyde product depends on the substrate [1]. The by-products of the reaction, hydrogen peroxide and ammonia, are invariably released.

In *Escherichia coli* (*E*. *coli*), a copper amine oxidase (ECAO) is encoded by the *tynA* gene (Entrez Gene ID: 945939, previously known as *maoA*, [2]). ECAO is a soluble, periplasmic, homodimeric protein with one copper ion and post-translationally derived topaquinones at both of its active sites [3]. Aromatic amines are the preferred substrates of ECAO, as compared to aliphatic amines and polyamines; therefore, it is also called 2-phenylethylamine oxidase or tyramine oxidase (http://www.expasy.org, [3]). ECAO was originally identified in *E*. *coli* K-12 [4], and its expression is detected only when the cells are growing in a medium supplemented with tyramine or phenylethylamine (PEA); it is not expressed under anaerobic conditions [5]. The expression of ECAO is regulated by FeaR, an AraC family regulator that also activates a phenylacetaldehyde dehydrogenase–encoding gene, *feaB* [5]. Because a transposon inserted into the *tynA* gene causes a constitutive SOS response [6], which protects against severe DNA damage caused by ultraviolet light or DNA-damaging chemicals [7], it has been suggested that *tynA* could be involved in catabolizing toxic compounds [8].

The crystal structure of ECAO has been solved [9]. It is a prototypic homodimer that has a catalytic section consisting of the D2–D4 domains and an additional D1 domain that is absent from other solved copper amine oxidase (CAO) structures. Although ECAO (as well as PrAOs from *Arthrobacter globiformis* and *Hansenula polymorpha*) has been used as a model enzyme to elucidate the enzymatic mechanism of amine oxidases [10–17], its known biological functions have so far been limited to enabling the bacteria to grow on different carbon or nitrogen sources [5,9,18]. In mammals, the corresponding primary amine oxidase, hAOC3, is involved in processes such as leukocyte trafficking and glucose metabolism [19].

In this study, we determined how widely this amine oxidase is found within the bacterial kingdom and in what circumstances it is expressed. We assessed ECAO’s biological role by performing microarray analysis on a deletion strain of *E*. *coli* K-12, Δ*tynA*. We found that ECAO can utilize a human leukocyte cell surface molecule(s) as a substrate and is inhibited by a hAOC3 inhibitor, which suggests that it shares similarities to its human counterpart. These results broaden our knowledge about this enzyme in bacteria and provide us with new insights into its role in *E*. *coli*.

## Materials and Methods

### Reagents

All the reagents were from Sigma-Aldrich unless otherwise stated.

### Bacterial strains and DNA

The *E*. *coli* strains used were DH5α [20], SY327 λ_pir_ [21], S17-1 λ_pir_ [22], and wild-type (wt) *E*. *coli* strains isolated from different environmental and human sources. Extended-spectrum beta-lactamase (ESBL) strains and wastewater samples were obtained from the National Institute for Health and Welfare, Turku, Finland. Bacterial pathogens were obtained from the Department of Microbiology, University of Turku, Finland. The DNAs of Finnish enterohemorrhagic *E*. *coli* (EHEC) strains were from the National Institute for Health and Welfare, Helsinki, Finland. The strains of different *E*. *coli* pathotypes were from the Institute for Molecular Infection Biology, University of Würzburg, Germany. *E*. *coli* strains isolated from well waters were obtained from the Finnish Water Protection Association, South-Western Finland, Turku, Finland. Strains from natural gut microbiota were collected from the fecal samples of healthy volunteers, and were only used for the development of the cultivation method. Stool samples analyzed for *tynA* and ECAO activity were from routine clinical microbiology laboratories. The vector pUC19 was from Fermentas (Waltham, MA, USA). *E*. *coli* (pKK233-3-tynA) [11] and pRV1 [23] have been characterized before.

### The presence of semicarbazide-sensitive amine oxidases in various bacteria

The presence of PrAO enzymes in bacteria was studied using two strategies: bioinformatics and functional assays. In the bioinformatics approach, we searched for bacteria with PrAO enzymes by querying published bacterial sequences (NCBI The Entrez Protein database) for established PrAO PROSITE motifs, namely a topaquinone signature (PS01164, LVVrwisTvgNYDY) and a copper-binding site signature (PS01165, TtGttHVaraEEwP). In the functional assay, we cultured 85 different *E*. *coli* strains in minimal medium with a specified amine as the only carbon source and tested the bacterial lysate for the presence of *tynA* by polymerase chain reaction (PCR, see below).

### Culture conditions

Bacteria were cultured in lysogeny broth (LB, [24]) or on Luria agar plates (LA) supplemented with the appropriate antibiotics (ampicillin, 25 μg/mL; chloramphenicol, 20 μg/mL; kanamycin, 100 μg/mL in the plates and 20 μg/mL in liquid media). Selected strains were screened for the expression of *tynA* by culturing the bacteria in an M9 minimal medium [25] supplemented with 2 g/L lactose and 5 mM PEA (M9-lactose-PEA medium). To induce ECAO activity, an overnight culture of wt *E*. *coli* in M9-lactose medium was diluted 1:20 in M9-lactose-PEA with 5 μM CuSO_4_. The bacteria were grown aerobically at 30°C with 250 rpm shaking. The induction of ECAO was assayed by measuring its activity (see below). To overexpress ECAO, we cultured *E*. *coli (*pKK233-3-tynA) in LB supplemented with 5 μM CuSO_4_ at 37°C and 250 rpm shaking to early log phase and induced the expression of ECAO by adding isopropyl β-1-D-thiogalactopyranoside (IPTG) to a final concentration of 2 mM.

### PCR screening for *tynA*

For the *tynA* screening, we took *E*. *coli* samples from fresh LA plates, suspended the bacteria in water, and incubated the samples for 5 min at 95°C. A total of 1 μL of bacterial lysate was used for PCR. We used forward primer EAO1 (5`-gacggggcatctttaccac-3`) and reverse primer EAO2 (5`-cacgccgctgaccgtagg-3`) to detect a 500–base pair (bp) fragment of the *tynA* gene. LACYfor (5`-ggtacttcaaacatatgcagcg-3`) and LACYrev (5`-ggctacatgacatcaaccatatc-3`), which amplify a 900-bp fragment of the *E*. *coli lacY* gene, were used as positive controls. Primers were from Operon (Cologne, Germany), and the Dynazyme polymerase was from Thermo Scientific (Espoo, Finland). The reaction products were subjected to agarose gel electrophoresis (Qbiogene QA-agarose, Qbiogene, Montreal, Canada), stained with ethidium bromide (Bio-Rad, Hercules, CA, USA), and visualized under ultraviolet light.

### Sequencing *tynA*

The *tynA* genes from three *tynA+* strains that grew in the M9-lactose-PEA medium and five *tynA+* strains that did not grow (indicating the enzyme was inactive) were cloned into the pCDNA3.1 plasmid and sequenced (Turku Centre for Biotechnology, University of Turku and Åbo Akademi University, Finland, primer sequences are available on request).

### *tynA* in the *E*. *coli* genome

The genomes of *tynA+* and *tynA-* strains were compared using the Artemis Comparison Tool (ACT) online (http://www.webact.org/, [26]).

### Construction of a *tynA* knockout strain (Δ*tynA*)

The *tynA* gene and 600 bp of flanking sequences on either side were amplified by PCR, using *E*. *coli* K-12 as a template, with the forward primer 5`- GTCAGA**GAATTC**CACATATGGATAAGATTGCTG -3`(EcoRI site in bold) and the reverse primer 5`- TCTGAC**GGATCC**GACACGCTGGTTAGTGG -3`(BamHI site in bold). The primers were designed according to the published sequence of the *tynA* locus in the *E*. *coli* genome [27]. The 3740-bp PCR product was cloned into the EcoRI/BamHI site of pUC19. A *tynA* fragment of 2250 bp was removed from the plasmid by inverse PCR (forward primer 5`-CTCAAG**ATGCAT**TCAGGTTGTTTTACGGGCAGAATAC-3`and reverse primer 5`-GTCAGA**ATGCAT**GACGAAACGCCAACGCTAG-3`, NsiI sites in bold). The *tynA* gene was replaced with a 1200-bp kanamycin resistance cassette (kmGB), which was a PstI cleavage product from the pUC-4K plasmid (GE Healthcare, Little Chalfont, UK). The *tynA* **Δ**kmGB was excised from pUC19 using PvuII, and the blunt ends were ligated into the EcoRV sites of the pRV1 suicide vector. The restriction enzymes EcoRI, BamHI, and NsiI were from NEB (New England Biolabs, Beverly, Massachusetts, USA), and EcoRV and PvuII were from Promega (Madison, Wisconsin, USA). Digestion products were purified from an agarose gel using the Qiagen Gel Extract Kit (Hilden, Germany), and the T4-ligation kit (NEB) was used for ligations.

The pRV1 vector containing the *tynA* knockout cassette (pRV1-TK) was electroporated first into SY327 λ_pir_ and then into S17-1 λ_pir_. The plasmid was conjugated from S17-1 λ_pir_ into wt ESBL *E*. *coli* 201. The transconjugant bacteria, now having the suicide vector integrated into their genomes by homologous recombination, were subjected to a cycloserine enrichment to select for clones resistant to kanamycin and ampicillin, and sensitive to chloramphenicol. In these clones, a second homologous recombination event had eliminated the suicide vector and the wild-type allele. The constructed mutants were verified by PCR using the primers 200bpFS (5`-GCAACGGCGAATAGTTGC-3`) and 200bpFSrev (5`-CGGTGTGGGTAAACAGCC-3`), and by Southern blotting.

### Southern blotting

Genomic DNA from wt and *ΔtynA E*. *coli* strains was isolated using the NucleoSpin Tissue Kit (Macherey-Nagel, Dueren, Germany). DNA was digested with HindIII (NEB), and Southern blotting was performed as described previously [28]. In the wt, the 3400-bp fragment encompassing *tynA* and 600-bp of flanking sequences was labeled using [α-32P]dCTP (50 μCi, 3000 Ci/mmol), according to the Prime-a-Gene Labeling System (Promega, Madison, WI, USA). The probe was purified using a ProbeQuant G-50 Micro Column, and the hybridization was performed in ULTRAhyb hybridization buffer (Ambion, Foster City, CA USA) at 42°C for 14–16 h. Probe detection was performed using X-ray film. The exposure time was 3 h.

### Preparation of cell lysates

Bacterial cultures were centrifuged at 3200 × *g* at 4°C for 15 min, and cell pellets were lysed in phosphate-buffered saline (PBS) containing 1 mM phenylmethylsulfonyl fluoride (PMSF), 1% aprotinin, and 0.1% nonyl phenoxypolyethoxylethanol (NP-40). The cells were lysed by three freeze-thaw cycles using liquid nitrogen and a 37°C water bath. The crude lysates were further treated with short ultrasound pulses (2 × 15 s).

### Determination of protein concentration

The concentration of protein within the lysates was determined using the Bio-Rad DC Protein Assay, with bovine serum albumin as the standard. The concentration of a purified protein was determined by a Bradford assay (Bio-Rad).

### Amine oxidase activity assay

To determine the amine oxidase activity in the lysates, we utilized the Amplex Red assay (Molecular Probes Europe BV), using 10 μg of bacterial lysate per well. PEA (0.5 mM) was used as a substrate. To specifically determine semicarbazide-sensitive amine oxidase activity, we used 1 mM semicarbazide (SC) in the control wells. ECAO activity was measured as the difference in fluorescence between SC-inhibited and uninhibited samples. To test two new substrates, isoamylamine and propylamine, we used the same assay and identical amounts of substrate. The activity assays were performed at 30°C.

To test if ECAO has a substrate on human granulocytes, we used purified recombinant protein ECAO, whose production and purification has been described [9]. To collect human neutrophils, we extracted granulocytes from the fresh peripheral blood of healthy volunteers using Ficoll (GE Healthcare). We collected buffy coats according to standard procedure, lysed red blood cells with Pharm Lyse (BD Biosciences, Franklin Lakes, NJ, USA), washed with PBS, counted the cells, and used the cells immediately. The assay was conducted in 25 mM HEPES and 150 mM NaCl (pH 7.4), and we used 30 nM ECAO and 200,000 viable granulocytes per well. PEA was used as a positive control for the substrate, and the specific activity was determined using parallel wells with SC, as described above. The assay was conducted at 37°C. Three independent assays (different blood donors) were performed with duplicates.

### ECAO inhibitor tests

To determine the effect of different inhibitors on ECAO activity, we used purified ECAO protein and a spectroscopic amine oxidase activity assay, which is not as sensitive to additives as the Amplex Red assay [29]. In short, 21 ng of ECAO protein was incubated with differing amounts of inhibitors in Krebs-Ringer phosphate glucose buffer for 20 min at 30°C. After incubation, we added substrate (0.5 mM PEA) and a mixture of 1 mM vanillic acid, 0.5 mM 4-aminopyridine, and 16 U of horseradish peroxidase, and measured the A_492nm_ immediately and hourly thereafter.

To assess the effect of inhibitors on bacterial growth, discs with different concentrations of SC were placed on LA plates freshly plated with either *tynA+ E*. *coli* (three different strains) or *tynA- E*. *coli* (five strains). The inhibition zones were measured the next day. For the growth inhibition assay in a liquid medium, the same strains were cultured in tryptic soy broth (TSB) medium for 14–16 h. Then, 10^3^ cells were inoculated into TSB medium with different concentrations (0–100 mg/mL) of SC and cultured further at 37°C with shaking at 100 rpm until the following day. Then, 10 μL of bacterial mixture was plated on lactose medium plates, and the plates were incubated at 37°C for 14–16 h before counting the colonies.

The inhibition of growth by a selective hydrazine-based human amine oxidase inhibitor, BTT-2052 [30], and SC on *tynA+* and *tynA-* strains was tested similarly in M9-lactose-PEA medium using inhibitor concentrations of 0–0.5 mM.

### RNA extraction

To extract RNA from the bacteria cultured in M9-lactose-PEA medium, we added two volumes of RNA Protect Bacteria (Qiagen) immediately to samples taken from the *E*. *coli* cultures at 0 h, 1 h, and 4 h. Total RNA was isolated using the RNeasy Mini-Kit (Qiagen), and DNA was removed using the RNase-Free DNase Set (Qiagen). The concentration of the extracted RNA was determined by a NanoDrop 2000 (Thermo Scientific), and the quality of RNA was quantified by Bio-Rad’s Experion electrophoresis station.

### cDNA preparation and array hybridization

Microarray studies were performed at the Finnish DNA Microarray Centre at Turku Centre for Biotechnology, Turku, Finland. Standard microarray methods were used for cDNA synthesis, fragmentation, and end-terminus biotin labeling, and were based on Affymetrix protocols. Equal amounts of total RNA from seven different samples were pooled to yield the required 10 μg of starting material, and 2.4 μg of fragmented and labeled cDNA was used in the preparation of the hybridization cocktail. The cocktail was prepared according to the Midi format. Each sample was hybridized to the GeneChip *E*. *coli* 2.0 Array. A GeneChip Fluidics Station 450 was used to wash and stain the arrays, and a GeneChip Scanner 3000 with Autoloader was used to scan the arrays. Affymetrix GeneChip Command Console (AGCC) software version 1.0 was used to control the Fluidic Station and Scanner. CEL files were automatically generated by AGCC. After the hybridization, quality control was done with the Affymetrix Expression Console.

### Data analysis

All the microarray data analysis was done using the R language and environment for statistical computing and Bioconductor [31,32]. The array data was normalized with the gcRMA method [33]. The resulting data was filtered using a threshold of >2.5 and <-2.5 for the fold-change of up- and down-regulated genes, respectively. The microarray data discussed here has been deposited in NCBI's Gene Expression Omnibus [34] and are accessible through GEO Series accession number GSE65385 (http://www.ncbi.nlm.nih.gov/geo/query/acc.cgi?acc=GSE65385).

### Production of hydrogen peroxide in the medium by ECAO

Wt ECAO was induced in M9-lactose-PEA medium cultures as described above. The *ΔtynA* strain was used as a negative control, and the cultures were grown for 6 h, as described. The cells were separated by centrifugation (1.5 min at 15300 × *g*), and the supernatant was assayed for activity by measuring the amount of hydrogen peroxide in the medium using the Amplex Red assay and by measuring the fluorescence using a fluorometer (TECAN Ultra, Tecan, Zürich, Switzerland).

### Ethics

For the cultivation of *tynA+* strains, as well as for measuring ECAO activity, we used collections of bacterial specimens that are investigated in routine clinical microbiology laboratories in Finland. In 2006, when the experiments were done, no ethical approval for the usage of these bacterial samples was needed, as all of them were from anonymous donors. For the development of a method to cultivate specimens from stool under specific conditions, we collected a few stool samples from the authors, who gave their oral informed consent for the usage of their samples for this purpose. One of the authors (T.K.) collected, stored, and cultivated the samples anonymously. We did not collect any identifying information from the donors, and none of the experiments or results were ever connected to their identities.

### Statistical analyses

The data are shown as the mean ± the standard error of the mean (SEM). Statistical tests, unless otherwise mentioned, were conducted using the Student’s t-test, and a value of p < 0.05 was considered statistically significant.

## Results

### Amine oxidases in various bacteria

To analyze which bacteria could harbor a homolog of PrAO, we conducted a database search using two PROSITE motifs that defined the active sites and the copper-binding sites of amine oxidases. The database search revealed that the amine oxidase gene exists in many environmental bacterial species (isolated from soil, sea water, pesticide- and metal-contaminated soil), as well as in opportunistic pathogens, but it exists in only a few clinically important species (S1 Table).

### ECAO in *E*. *coli*

The existence of *tynA* in different *E*. *coli* strains was assayed first by screening 38 Finnish wastewater and well water samples for the presence of *tynA* using PCR (Table 1, S1 Fig). More than half of the well water samples (57%) and about one third of the wastewater samples (35%) were *tynA+*. The subsequent screening of different human isolates and *E*. *coli* pathotypes indicated that this gene could be frequently detected in diarrhea-causing pathotypes, such as non-O157:H7 EHECs (82% *tynA+*), enterotoxigenic *E*. *coli* (ETEC; 69%), and enteroinvasive *E*. *coli* (EIEC; 67%), as well as in fecal isolates (38%, Table 2). Of note, *tynA* was less frequently detected in enteropathogenic *E*. *coli* (EPEC; 17%). Among extra-intestinal pathogenic isolates, *tynA* was most frequently detected in sepsis isolates (27%). Out of the few tested pus (7), wound (3), and blood (8) samples, 71%, 67%, and 50% were *tynA+*, respectively. The *tynA* screening was negative in newborn meningitis (11) and in O157:H7 EHEC (28) isolates.

**Table 1:** Frequency of *E*. *coli* in environmental isolates.^a^

	Waste water	Well water
Total	20	18
*tynA+*	7	12
%	35	57

^a^tested by PCR.

**Table 2:** Frequency of *E*. *coli* in human isolates.^a^

	Wound	Pus	Blood	Sepsis	Feces	EIEC	EPEC	UPEC	ETEC	NBM	EHEC O157:H7	EHEC Non-O157:H7
Total	3	7	8	15	99	3	6	45	13	11	28	49
*tynA+*	2	5	4	4	38	2	1	3	9	0	0	40
%	67	71	50	27	38	67	17	7	69	0	0	82

^a^tested by PCR.

### Genome comparisons

To gain further insight into the evolution of *tynA* in *E*. *coli*, we compared the genomes of a *tynA-*positive (K-12) and a *tynA*-negative (UPEC CFT073) strain with the ACT [26], which revealed that a 33-kb area flanking the *tynA* gene is missing from CFT073 (S2 Fig). When another *tynA*-negative strain, IHE3034, was included, we found the rearrangement of 930 kb of flanking sequence by an inversion.

### Natural *tynA* mutations

When screening for culture conditions that induced *tynA* expression, we found that ECAO activity was induced only when *tynA+* strains were grown with PEA as the carbon source (data not shown). However, when we tested the amine oxidase activity of several strains that were *tynA+*, according to our PCR screening, some did not grow in M9-lactose-PEA medium. Only a subset (22 out of 39 *tynA+* strains) both grew and had amine oxidase activity, as determined in our standard Amplex Red assay (S2 Table). The sequencing of *tynA* genes from seven strains, both inactive and active, demonstrated that four strains, including DH5α, had mutations in *tynA* when compared to the *tynA* of wt *E*. *coli* K-12, but none of the mutations were in the conserved residues essential for amine oxidase activity (S3 Table). Therefore, we propose that the presence of *tynA* does not necessarily reflect the existence of the functional enzyme.

### ECAO expression and activity depends on the presence of both lactose and PEA in the medium

To study the biological role of *tynA* in *E*. *coli* K-12, we constructed a *tynA*-deletion strain (Δ*tynA*) through allelic exchange. The successful allelic exchange was verified by PCR, and the deletion of *tynA* was confirmed by Southern blot (S3 Fig).

**Fig 1:**
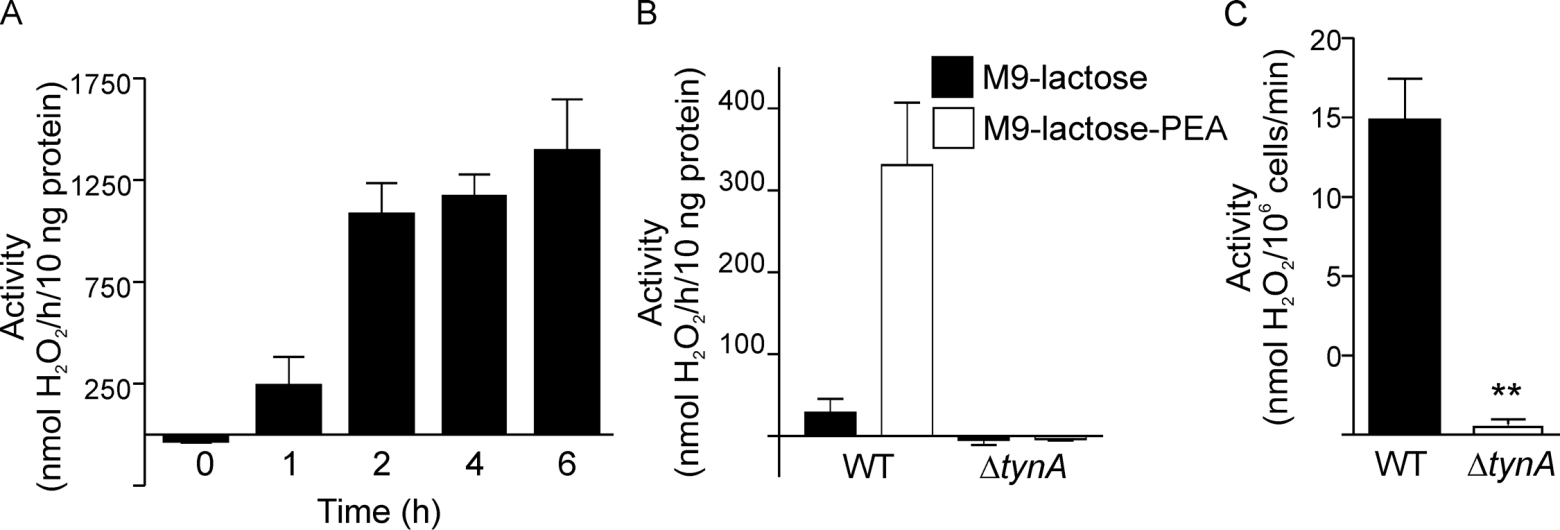
The time and medium dependence of ECAO expression and activity. **(**A) The kinetics of ECAO induction in *E*. *coli* K-12 (wt) cultured in M9-lactose medium supplemented with 5 mM PEA (n = 4). The 0 h time point corresponds to the time when the cells were diluted into PEA-containing culture medium. (B) The activity of ECAO is induced in *E*. *coli* K-12 (wt), but not in our Δ*tynA* strain, by the presence of both lactose and PEA 6 h after diluting the bacterial cultures in M9-lactose-PEA media (n = 3). (C) The H_2_O_2_ production of wt K-12 *E*. *coli* and Δ*tynA* bacteria in stationary phase in M9-lactose-PEA medium (** *p* < 0.01). The means ± SEMs are shown.

We assayed the expression and activity of the *tynA* gene product, ECAO, on PEA in different media. When culturing wt bacteria in M9-lactose-PEA medium, we detected activity on PEA (5 mM) after 1 h, and the activity reached a maximum at 2 h after induction (Fig 1A). No significant activity was detected in M9-PEA medium supplemented with another sugar, such as glucose, arabinose, rhamnose, sucrose, or maltose, as a carbon source (data not shown). To analyze the relationship between ECAO expression and the presence of both lactose and PEA in the medium, we tested the amine oxidase activity of wt and Δ*tynA* strains in minimal medium M9 supplemented with lactose, with or without 5 mM PEA. We detected a clear dependence of activity on the presence of lactose and PEA for wt, but the Δ*tynA* strain had no activity in either (Fig 1B), which has been shown before with a different medium [18]. To measure the amount of H_2_O_2_ released into the medium by ECAO, we measured the H_2_O_2_ concentrations of stationary phase cultures of wt and Δ*tynA* strains. Again, the Δ*tynA* strain failed to produce any H_2_O_2_ in the medium, whereas the wt strain had concentrations of up to 1 μM H_2_O_2_ (Fig 1C), similar to earlier findings [18]. To test the endurance of ECAO expression, a total of nine different strains (and our Δ*tynA* strain) were cultured in similar conditions for 7 d, during which the cells continued to grow without entering stationary phase. ECAO activity remained at a similar level, although it differed among the strains. The Δ*tynA* strain was constantly inactive (data not shown).

### Inhibition of ECAO with semicarbazide

**Fig 2:**
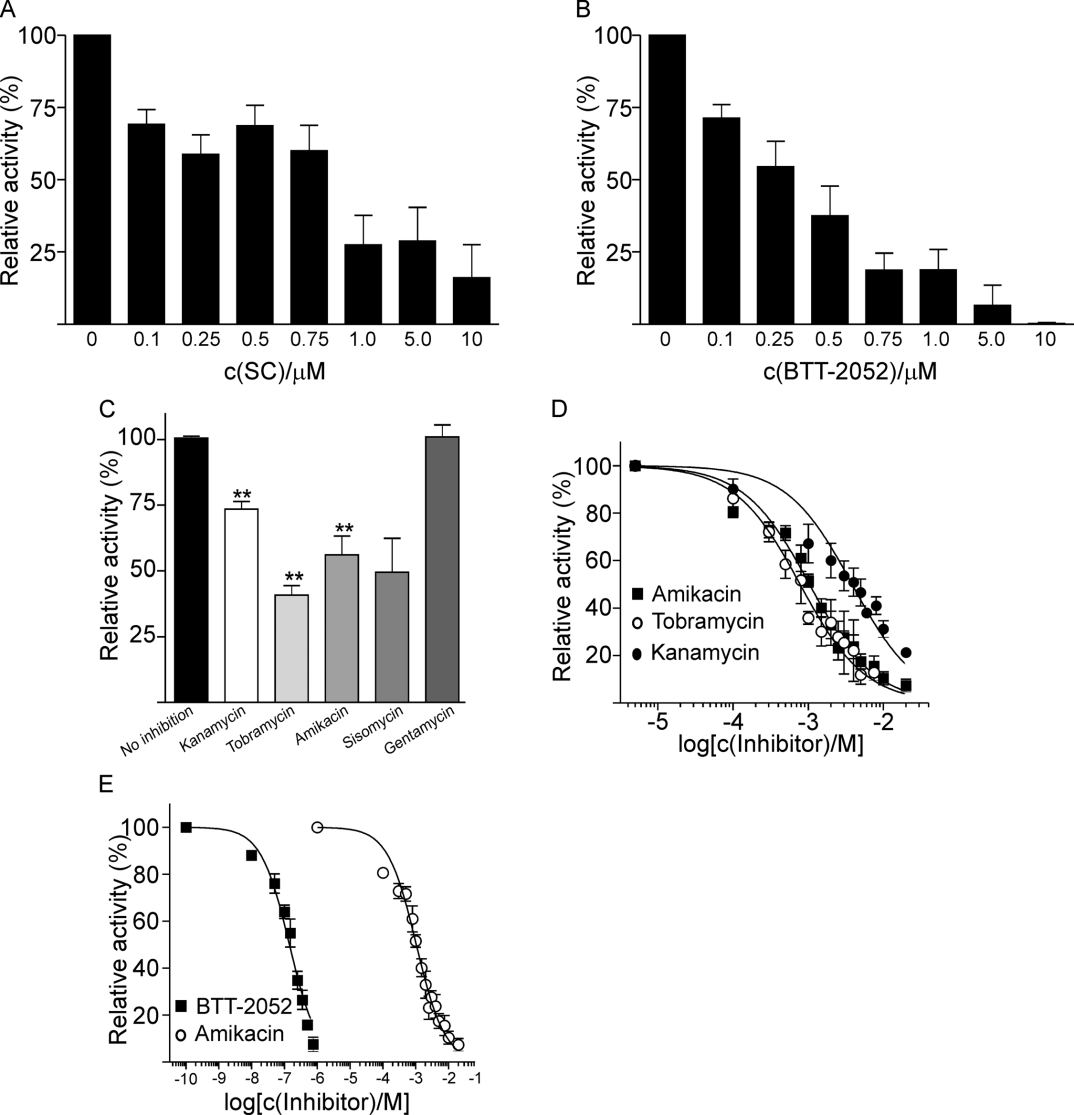
Different ECAO inhibitors. (A) The relative inhibition of ECAO by semicarbazide (SC) and (B) the hAOC3 inhibitor BTT-2052. (C) The effect of different aminoglycosides (1 mM) on the activity of ECAO (** *p* < 0.01). (D) The inhibition curves of different aminoglycosides on ECAO activity. (E) The pairwise comparison of inhibition curves between BTT-2052 and amikacin.

ECAO preferentially processes aromatic amine substrates such as PEA, tyramine, and tryptamine, whereas the human homolog of ECAO, hAOC3, is most active towards benzylamine (BA) and methylamine [3,35]. In our experiments, we confirmed the preferential activity of ECAO on PEA, tyramine, and tryptamine over BA (S4 Fig). To our knowledge, the effects of different amine oxidase inhibitors on ECAO have not been tested; therefore, we compared the effects of a general PrAO inhibitor, SC, and a selective hAOC3 inhibitor, BTT-2052, on ECAO activity on PEA (Fig 2). Among seven different inhibitor concentrations (0.1–10 μM), we observed a half maximal inhibitory concentration (IC_50_) of 0.7 μM for SC and 0.24 μM for BTT-2052.

We tested the effect of the SC and BTT-2052 inhibitors on the growth of the wt and Δ*tynA* strains on plates and in liquid media. When we used SC concentrations lower than 5–10 mM, we detected no significant effect on growth in liquid rich media (S5 Fig). As expected, SC had no effect on the growth of the Δ*tynA* strain (S5 Fig). On LA plates, SC had no effect on bacterial growth under the concentration of 100 mg/mL, and even at 100 mg/mL, the effect was minimal (data not shown). BTT-2052 had no effect on the growth of either wt or Δ*tynA* strains on plates at the investigated concentrations (0.1–0.5 mM, data not shown).

### Certain aminoglycosides are able to inhibit ECAO activity

Aminoglycosides, which consist of branched primary amine groups attached to a ring structure (tetrahydropyran or cyclohexane), are antibiotics against Gram-negative bacteria and are reported to inhibit bovine amine oxidase [36]. We tested the effect of aminoglycosides on ECAO activity; kanamycin, tobramycin, amikacin, and sisomycin (*p* = 0.06) decreased activity by 23–61% (Fig 2C). Gentamycin had no effect. To quantify the inhibition kinetics of amikacin, tobramycin, and kanamycin, we measured the activity of ECAO on PEA in different aminoglycoside concentrations (10 μM–10 mM). The half maximal inhibitory concentrations (IC_50_) were 1 mM for amikacin, 0.8 mM for tobramycin, and 3.8 mM for kanamycin (Fig 2D). The inhibition kinetics of amikacin and BTT-2052 were compared across nine different inhibitor concentrations (Fig 2E). The shape of the curves is similar, but the IC_50_ values differ by an order of magnitude.

### Knocking out *tynA* interferes with several pathways in *E*. *coli*

**Fig 3:**
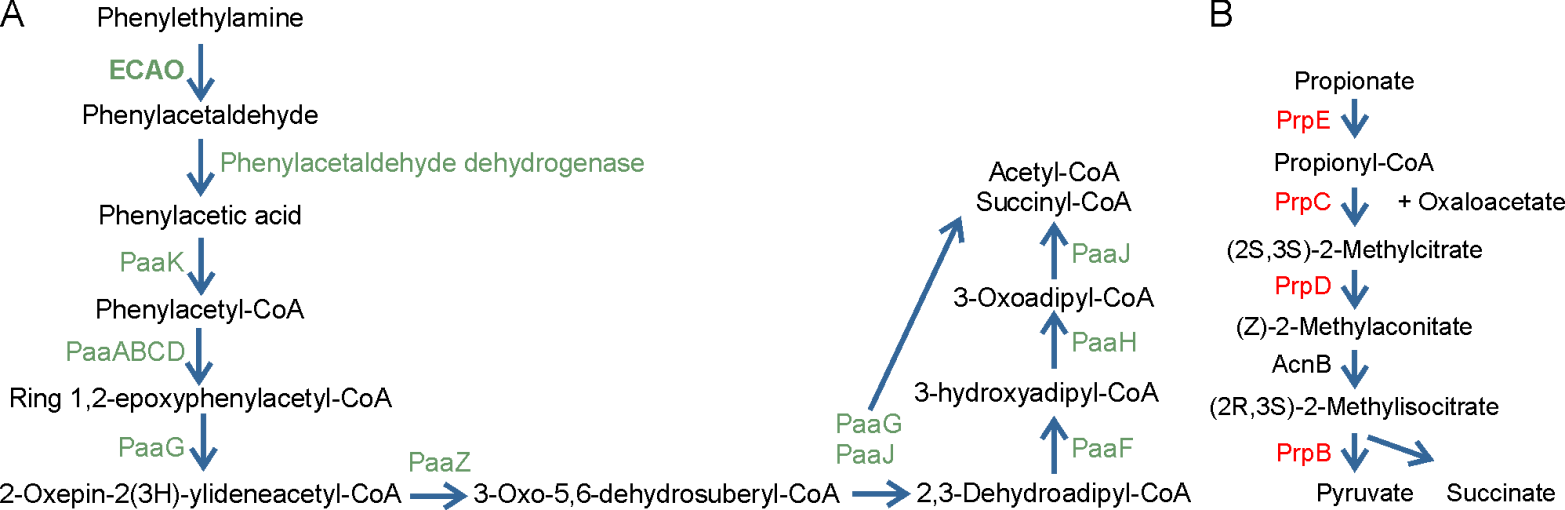
Pathways altered by *tynA* knockout. **(**A) The phenylethylamine metabolic pathway is down-regulated (green) in the Δ*tynA* strain when compared to the wt strain in M9-lactose-PEA medium. (B) The oxidation of propionate to pyruvate is up-regulated (red) in the Δ*tynA* strain. Pathways are depicted according to Brock *et al*. and Zeng *et al*. [5,41].

To assay the biological role of *tynA* in *E*. *coli*, we performed microarray analysis using RNA from wt and Δ*tynA* cell lysates. After 4 h of growth in M9-lactose-PEA medium, the most differentially expressed gene between Δ*tynA* and wt was *tynA*, as expected (214-fold down-regulated, Table 3). In addition, the enzymes involved in the aerobic catabolic pathway of phenylacetate (*paaABCDEFGHIJKXY* and *maoC* [*paaZ*]) and phenylacetaldehyde dehydrogenase (*feaB*) were down-regulated (FC = -108 –-2.9, Fig 3A). Furthermore, we noticed a decrease in the expression of *oxyS*, *grxA*, and *ahpCF*, which are regulated by the oxidative stress regulator OxyR [37]; OxyS is a regulatory sRNA, GrxA is a glutaredoxin, and AphCF is an alkyl hydroperoxide reductase [38–40]. Other genes, which were expressed with less than a -2.5-fold difference, were hypothetical proteins *c0693* and *c4090*, and S-adenosyl-L-methionine-dependent methyltransferase (*smtA*, FC -2.8, -2.8, and -2.9, respectively).

**Table 3:** Differentially down-regulated (FC < -2.50) genes in Δ*tynA*.^a^

Gene symbol	Gene	FC
***tynA***	**Tyramine oxidase, copper-requiring**	**-213.77**
***pad***	**Predicted multicomponent oxygenase/reductase subunit for phenylacetic acid degradation**	**-108.30**
***paaC***	**Predicted multicomponent oxygenase/reductase subunit for phenylacetic acid degradation**	**-86.95**
***maoC***	**Fused aldehyde dehydrogenase/enoyl-CoA hydratase**	**-83.89**
***paaA***	**Predicted multicomponent oxygenase/reductase subunit for phenylacetic acid degradation**	**-76.72**
***paaB***	**Predicted multicomponent oxygenase/reductase subunit for phenylacetic acid degradation**	**-67.16**
***paaG***	**Acyl-CoA hydratase**	**-55.67**
***paaE***	**Predicted multicomponent oxygenase/reductase subunit for phenylacetic acid degradation**	**-53.42**
***paaF***	**Enoyl-CoA hydratase-isomerase**	**-49.38**
***feaB***	**Phenylacetaldehyde dehydrogenase**	**-39.84**
***paaI***	**Predicted thioesterase**	**-24.85**
***paaJ***	**Predicted beta-ketoadipyl CoA thiolase**	**-22.80**
***paaK***	**Phenylacetyl-CoA ligase**	**-13.82**
***paaH***	**3-Hydroxybutyryl-CoA dehydrogenase**	**-13.09**
***oxyS***	**OxyS RNA**	**-10.16**
***grxA***	**Glutaredoxin 1**	**-6.30**
***paaX***	**DNA-binding transcriptional repressor of phenylacetic acid degradation, aryl-CoA responsive**	**-4.81**
***smtA***	**Putative metallothionein**	**-2.88**
***pay***	**Predicted hexapeptide repeat acetyltransferase**	**-2.87**
***ahpF***	**Alkyl hydroperoxide reductase subunit F**	**-2.85**
***c4090***	**Hypothetical protein**	**-2.79**
***ahpC***	**Alkyl hydroperoxide reductase subunit C**	**-2.76**
***c0693***	**Hypothetical protein**	**-2.75**

^a^Comparisons to wt bacteria were made at the 4 hr time point

The most up-regulated genes in Δ*tynA* were those encoding the enzymes involved in oxidizing propionate to pyruvate: *prpBCDE* with FC 9.38 to 3.39 (Table 4, Fig 3B). Notably, there were fewer genes up-regulated than down-regulated, and the magnitude of up-regulation was lower (the highest fold-change was 9.38, whereas the lowest was -213.77). Two other pairs of up-regulated genes were those of the nitrogen regulatory protein C (NtrC)–activated polar amino acid transporter family *yhdXZ* [42], with FC = 6.09 and 3.44, respectively, and the respiratory nitrate reductase genes *narUW* [43], with FC = 2.97 and 3.14 respectively. See Table 4 for the other up-regulated genes.

**Table 4:** Differentially up-regulated (FC > 2.50) genes in *ΔtynA*.^a^

Gene symbol	Gene name	FC
***prpE***	**Putative propionyl-CoA synthetase**	**9.38**
***prpD***	**2-Methylcitrate dehydratase**	**8.50**
***prpC***	**Methylcitrate synthase**	**7.99**
***yhdX***	**Putative transport system permease protein**	**6.09**
***gabT***	**4-Aminobutyrate aminotransferase**	**3.53**
***yhdZ***	**Hypothetical amino-acid ABC transporter ATP-binding protein YhdZ**	**3.44**
***prpB***	**2-Methylisocitrate lyase**	**3.39**
***narW***	**Cryptic nitrate reductase 2 delta**	**3.14**
***narU***	**Nitrite extrusion protein 2**	**2.97**
***aldB***	**Aldehyde dehydrogenase B (lactaldehyde dehydrogenase)**	**2.83**
***pqiA***	**Paraquat-inducible protein A**	**2.71**
***hemN***	**Coproporphyrinogen-III oxidase**	**2.60**
***ygaM***	**Hypothetical protein**	**2.53**
***glgS***	**Glycogen synthesis protein GlgS**	**2.53**
***ldhA***	**D-Lactate dehydrogenase**	**2.50**

^a^Comparisons to wt *E*. *coli* were done at the 4 h time point.

### ECAO activity on granulocytes

**Fig 4:**
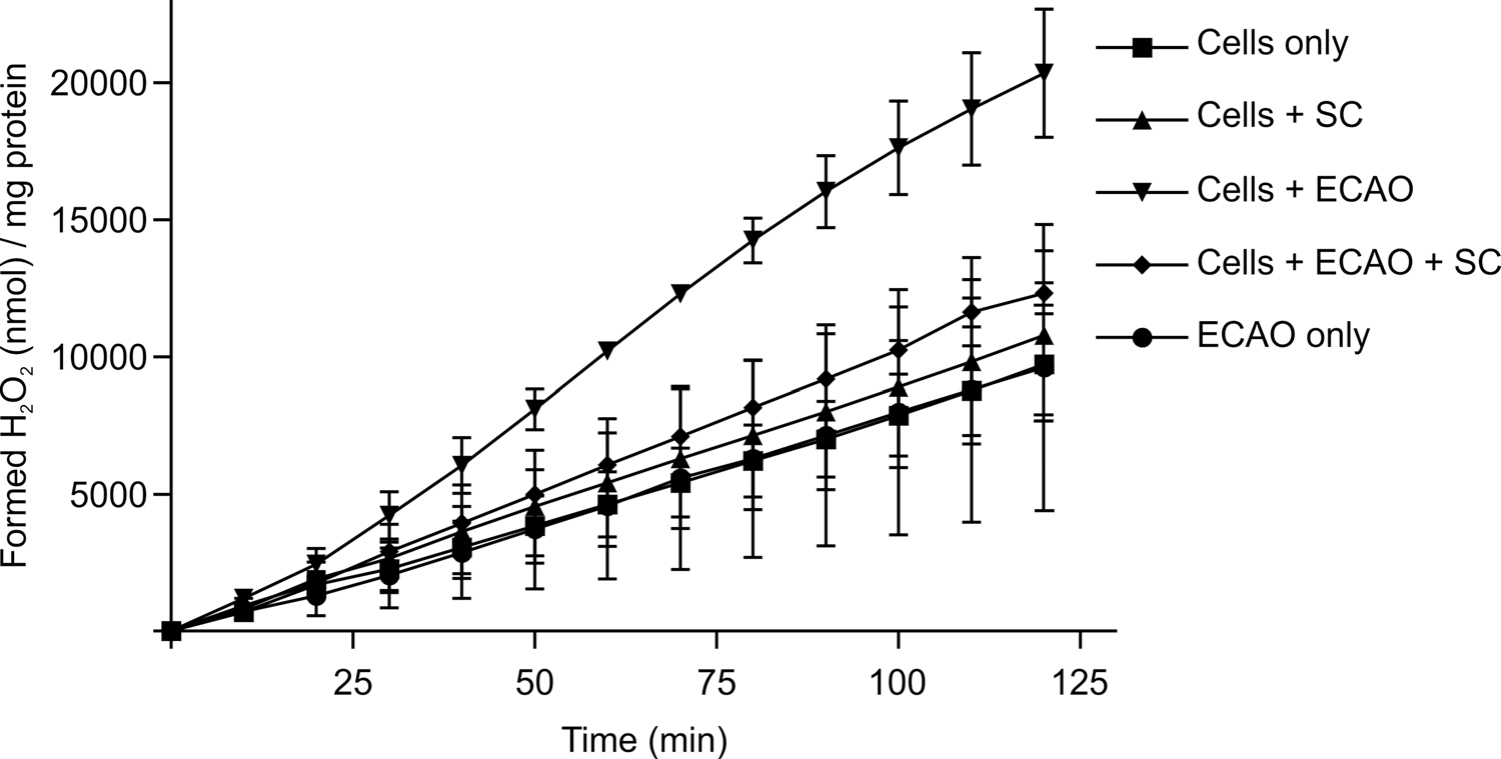
ECAO is able to use human granulocytes as a substrate. We detected the production of H_2_O_2_ by ECAO when granulocytes were the only source of substrate (Cells + ECAO), and the activity was inhibited by semicarbazide (Cells + ECAO + SC). The activity is significantly higher than without ECAO (Cells only, *p* = 0.0003; cells with SC, *p* = 0.0004; without cells, *p* = 0.017, and with added SC inhibitor [Cells+ECAO+SC], *p* = 0.004). The mean of three independent experiments is shown ± SEM. Statistical significance was calculated with paired t-test.

To determine whether bacterial ECAO could function like its human counterpart, hAOC3, by acting on human leukocytes, we tested the activity of purified, recombinant ECAO protein [9] with fresh human granulocytes as the sole source of substrate. We detected significantly more H_2_O_2_ production when granulocytes were added to ECAO than in the condition without cells, without ECAO, or with SC as an inhibitor (Fig 4). Clorgylin was added to prevent monoamine oxidase activity.

## Discussion

We found that the gene encoding the primary amine oxidase, *tynA*, is present in various bacteria. We screened several environmental and human isolates for both *tynA* presence and activity, and demonstrated that some strains possess the gene, but do not have its activity. We replicated the previous results that ECAO enables bacteria to grow on a rare nitrogen or carbon source, such as PEA, and that the activity of the *tynA* gene product in *E*. *coli* periplasm drives the production and release of hydrogen peroxide into the environment [18]. As new findings, we demonstrated that ECAO is inhibited by certain aminoglycosides and report that ECAO can utilize human leukocytes as substrates. In addition, ECAO was inhibited by a human amine oxidase inhibitor suggesting that the bacterial protein mimics the enzymatic activity of the human homolog.

### *tynA* is a reserve gene for harsh culture conditions

Our database search for the existence of *tynA* revealed that opportunistic pathogens, human and plant pathogens, antibiotic-producing bacteria, and bacteria resistant to antibiotics harbor an amine oxidase homolog of ECAO (S1 Table). The environmental bacteria included isolates from harsh conditions, such as the soil, pesticide- or metal-contaminated soil or seawater, suggesting that *tynA* serves as a reservoir gene for circumstances when nutrients are not abundantly available. This was supported by our detecting more *tynA-*positive *E*. *coli* strains in well water than in wastewater (Table 1). On the other hand, the release of hydrogen peroxide into the immediate environment may be bactericidal to those bacteria that do not express peroxidases, such as catalase in *E*. *coli* K-12 [44,45]. As has been shown previously [18], we also measured hydrogen peroxide at a concentration of 1 μM in the medium of *tynA*+ bacterial cultures in stationary phase, and this was *tynA* dependent (Fig 1). ECAO is a periplasmic enzyme and the hydrogen peroxide formed by its enzymatic reaction is secreted into the environment, because the flux of the formed hydrogen peroxide is larger through the outer membrane than through the inner membrane [18]. Even small amounts of hydrogen peroxide may give a growth advantage to ECAO-producing bacteria in their immediate surroundings.

### O157:H7 *E*. *coli* are *tynA*-negative

We observed the absence of *tynA* in serotype O157:H7 EHEC (Table 2). Some O157:H7 bacteria lose O157 antigenicity or survive poorly in water cultures [46,47], suggesting that growth conditions favoring the presence of *tynA* could be specifically useless or harmful for O157:H7 pathogenicity. In addition, PEA inhibits cell division and reduces O157:H7 *E*. *coli* counts in beef [48], which further supports our finding.

### ECAO processes aromatic amines and is affected by a human PrAO inhibitor and aminoglycosides

As a semicarbazide-sensitive amine oxidase, ECAO is able to process PEA, tyramine, and tryptamine, and its activity is inhibited by SC, hydroxylamine, phenelzine, iproniazid, and tranycypromine [3]. We found that the hAOC3 inhibitor BTT-2052 also inhibited ECAO with an efficient inhibitor concentration lower than that of SC (0.24 μM vs. 0.7 μM, respectively, Fig 2A and 2B). This is within the same range of IC_50_ values for recombinant hAOC3, mouse and rat AOC3, and purified porcine AOC1 (0.05–0.18 μM, [30]). In addition, the IC_50_ of BTT-2052 for ECAO was lower than that for mouse PrAO, hAOC2, and for recombinant human MAO-A [30]. We also demonstrated that the aminoglycosides tobramycin, amikacin, and kanamycin inhibited ECAO activity (IC_50_ of 0.8–3.8 mM, Fig 2C–2E), which may have some clinical relevance. Although observed IC_50_ concentrations are one order of magnitude higher than the maximum concentration recommended for these antibiotics in human serum (2–72 μM), concentrations 100 times higher than in serum can be locally achieved, as when antibiotics accumulate in the kidney during their excretion [49].

### ECAO is a metabolic enzyme

To analyze the role of ECAO in *E*. *coli* K-12, we performed a microarray analysis on the wt and Δ*tynA* strain. Most of the down-regulated genes in Δ*tynA* were either part of the *paaABCDEFGHIJKXYZ* operon, which produces enzymes to convert phenylacetic acid to acetyl-CoA and succinyl-CoA, or were related to it (*tynA* and *feaB*) and were active in utilizing PEA as a carbon source (Fig 3A, [50,51]). Another down-regulated group of genes were those under the control of OxyR (*oxyS*, *aphCF*, and *grxA*), which is present in its inactive, reduced form *in vivo* [52]. OxyR is activated by hydrogen peroxide, and once oxidized, drives the transcription of *oxyS*, *ahpCF*, and *grx*, among other genes [52,53]. Furthermore, Δ*oxyR* cells are unable to grow on PEA, but grow on phenylacetic acid [18]. Because we observed no down-regulation of the other members of the regulon [54], the down-regulation of *oxyS*, *aphCF*, and *grxA* most likely results from the activation of OxyR by the hydrogen peroxide produced by ECAO. In addition, there was no significant difference in the levels of the cyclic adenosine monophosphate (cAMP) or the cAMP regulatory protein (CRP) genes (*cyaA*, FC 1.21 and *crp* FC 1.11, respectively), despite *paaABCDEFGHIJK* and *paaZ* being regulated by CRP [55–57]. This is supported by the recent results that *paaXY*, which were both down-regulated in our work, form an auto-regulated operon, and PaaY regulates the transcription of other *paa* genes [58]. The roles of the rest of the down-regulated genes in Δ*tynA* remain to be elucidated.

In the Δ*tynA* strain, the *prpBCDE* operon was up-regulated. Its proteins are involved in degrading propionate to pyruvate in the 2-methylcitrate pathway ([41], Fig 3B). The *prpBCDE* operon is activated by carbon catalytic repression, which ensures the utilization of the most energy-rich sugar (glucose) in the growth environment via regulation by cAMP, CRP, and σ^54^ ([55,59–61]). We believe that when *tynA*+ *E*. *coli* strains are grown in PEA-lactose minimal medium, this operon is down-regulated, enabling the utilization of PEA. When PEA cannot be utilized by the Δ*tynA* strain, the repression of the operon is not active, and the genes are up-regulated compared to the wt strain. Previously, it was shown that *prpBCDE* is down-regulated by 2 g/L lactose in rich culture medium [62], and we found this is also true for minimal medium. The fourth enzyme involved in the 2-methylcitrate pathway, *acnB*, was only slightly up-regulated (FC 1.6), but it is expressed in all growth conditions, independent of the energy source [41]. Two other genes related to the propionate pathway were also up-regulated in Δ*tynA* cells: *ldhA*, whose gene product, lactate dehydrogenase, converts pyruvate to lactate [63] and *aldB*, which encodes for an enzyme that converts propanal to propionate [64].

Nitrogen starvation can induce *tynA* expression [5]. Therefore, the up-regulation of genes in Δ*tynA* could result from the absence of ammonia produced by ECAO in wt cells. Of note, *narWU* and *yhdXZ* genes were up-regulated in the Δ*tynA* strain. Both *yhdX* and *yhdZ* are activated by nitrogen limitation [42,65]. In addition, *narU*, which is involved in nitrate uptake [66], and *narW*, which is needed in the formation of active respiratory nitrate reductase complex NRZ from the *narZYWV* operon [43], were up-regulated. The NarY subunit of the complex contains an iron-sulfur cluster, and the formation of such clusters are catalyzed, in anaerobic conditions, by an oxygen-independent coproporphyrinogen-III oxidase, a gene product of *hemN* [67], which was also up-regulated in the Δ*tynA* strain. Moreover, *prpBCDE*, *yhdXZ*, and *hemN* are all under the control of the RNA polymerase subunit σ^54^, which is important in nitrogen assimilation [61].

Finally, *glgS* was also up-regulated. It encodes for a glycogen synthesis molecule that is induced during stationary phase and may be a significant repressor of bacterial adhesion and biofilm formation [68]. Its up-regulation in Δ*tynA* cells could simply be a consequence of more stringent culture conditions. In conclusion, the microarray analysis of up-regulated genes in the Δ*tynA* strain identified new genes involved in PEA-dependent nitrogen utilization and confirm the metabolic pathways affected by the lack *tynA*.

It has been known for a long time that PrAOs are involved in amine metabolism. It is an advantage for bacteria to be able to grow on different nutrients. For example, *Klebsiella oxytoca*, *E*. *coli*, and *Arthrobacteria* use oxidases to grow on, preferably, aromatic amines [3,69,70]. *Arthobacter globiformis* has two different oxidases, enabling it to grow on both PEA and histamine [71,72], and *Arthrobacter P1* is able to grow on methylamine [73]. This capability is not, however, restricted to bacteria. Yeasts have been proposed to use these enzymes in their metabolism [74,75]. Eukaryotic PrAOs, in general, have markedly different substrate specificity than prokaryotes, as they process BA and methylamine over other substances like tyramine or PEA [76–78], but their biological functions differ from those of prokaryotes [77,79].

### ECAO can use human granulocytes as a substrate

The human counterpart of ECAO, hAOC3, is involved in the adhesion and transmigration of leukocytes from blood vessels to the sites of inflammation [80]. hAOC3 utilizes lymphocyte surface molecules as a substrate, and Siglec-9 and -10 (Sialic acid–binding immunoglobulin-like lectins) are its leukocyte counter-receptors [81–83]. The activity of hAOC3 is important for its biological role in leukocyte trafficking [84]. Because ECAO is inhibited by a hAOC3 inhibitor and has a similar molecular structure, we tested ECAO’s activity on human leukocytes. Indeed, purified recombinant ECAO was able to process human granulocytes, as determined by the production of H_2_O_2_ (Fig 4), even though we and others have demonstrated different substrate specificities for these two proteins [3,76]. Although it is possible that purified PrAOs from other bacteria, plants, and yeast can also process molecules on human granulocytes, we demonstrated that *E*. *coli* ECAO behaves in a similar manner as hAOC3 under these experimental conditions, yet the *in vivo* relevance remains to be seen.

## Supporting Information

S1 Fig An example of the *tynA* screening by PCR. (DOCX)

S2 Fig ACT comparison of the *tynA*-positive K-12 and *tynA-*negative CF073 strains. (DOCX)

S3 Fig Construction of the Δ*tynA* strain. (DOCX)

S4 Fig The activity of ECAO-expressing cell lysates. (DOCX)

S5 Fig The effect of ECAO inhibition on bacterial growth and the production of hydrogen peroxide. (DOCX)

S1 Table Bacteria harboring the amine oxidase motifs. (DOCX)

S2 Table *tynA+ E*. *coli* strains tested for ECAO activity. (DOCX)

S3 Table ECAO mutations* found in *tynA+ E*. *coli* strains. (DOCX)
