# Supplementary material for: Primary Amine Oxidase of Escherichia coli Is a Metabolic Enzyme that Can Use a Human Leukocyte Molecule as a Substrate
Source: PLoS One. 2015 Nov 10;10(11):e0142367. doi: 10.1371/journal.pone.0142367 (PMC4640556; doi:10.1371/journal.pone.0142367)
Supplement: S1 Fig — (DOCX) [file pone.0142367.s001.docx]

**
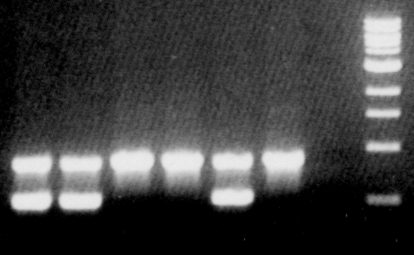
**

**S1 Fig. An example of the *tynA* screening by PCR.** The template DNAs were from *E. coli* strains isolated from fecal samples. The upper band is a 900 bp positive control (*lacY*) and the lower band is from the ECAO gene (*tynA*, 500bp). There are three *tynA+* (lanes 1-2 and 5 from left) and three *tynA-* samples (lanes 3-4 and 6). Negative control (lane 7), size marker (lane 8).
