# Supplementary material for: Primary Amine Oxidase of Escherichia coli Is a Metabolic Enzyme that Can Use a Human Leukocyte Molecule as a Substrate
Source: PLoS One. 2015 Nov 10;10(11):e0142367. doi: 10.1371/journal.pone.0142367 (PMC4640556; doi:10.1371/journal.pone.0142367)
Supplement: S2 Fig — (DOCX) [file pone.0142367.s002.docx]

**A**


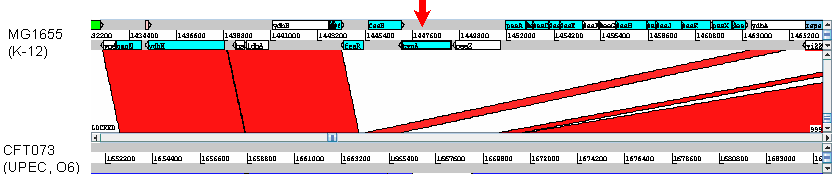


***tynA***

**B**


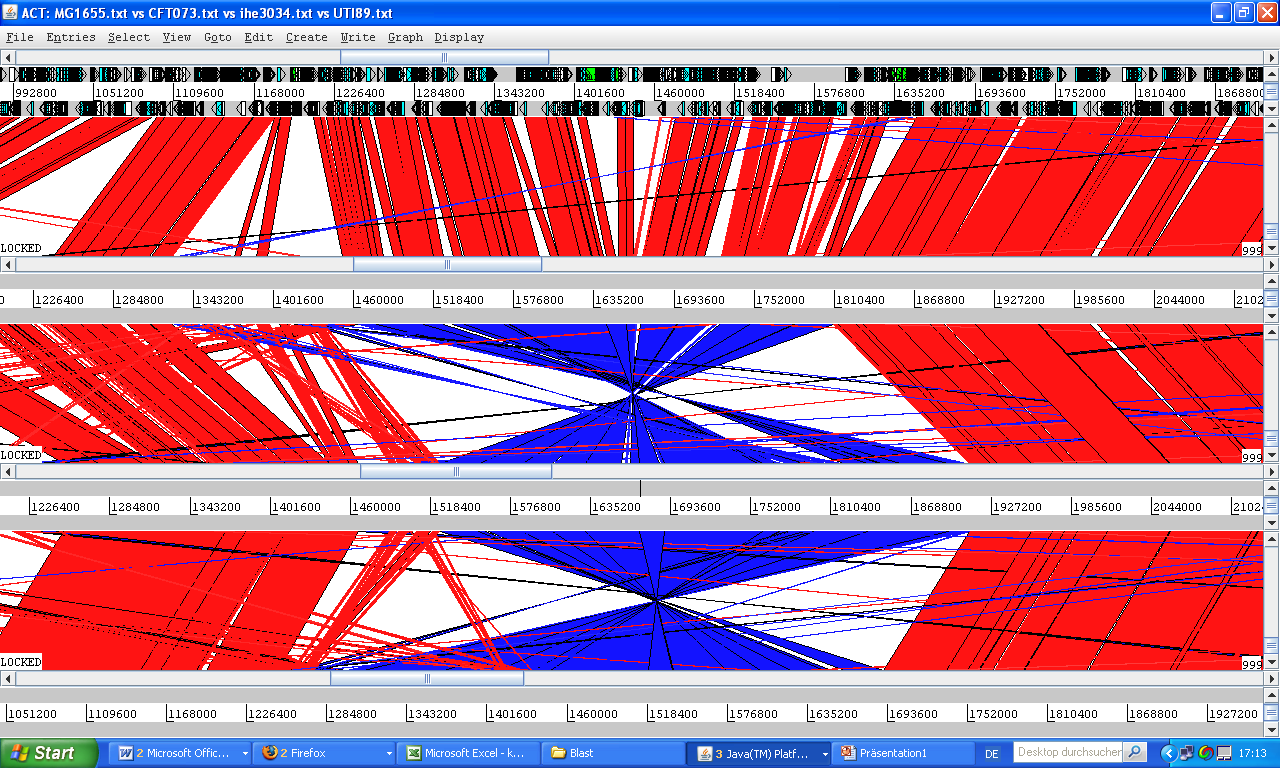


MG1655

(K-12)

CFT073

(UPEC, O6)

IHE3034

(MNEC, O18)

UTI89

(UPEC)

***tynA***

**S2 Fig. ACT comparison of *tynA* positive K-12 and *tynA* negative CF073 strains.** **A,** *tynA* and 33 kb sequence around it is absent from uropathogenic CFT073. **B,** ACT comparison of 930 kb around *tynA* in K-12 to *tynA-*negative CF073 and IHE3034. Red lines, similar sequences, and blue lines, inverted similar sequences.
