# Supplementary material for: Primary Amine Oxidase of Escherichia coli Is a Metabolic Enzyme that Can Use a Human Leukocyte Molecule as a Substrate
Source: PLoS One. 2015 Nov 10;10(11):e0142367. doi: 10.1371/journal.pone.0142367 (PMC4640556; doi:10.1371/journal.pone.0142367)
Supplement: S3 Fig — (DOCX) [file pone.0142367.s003.docx]

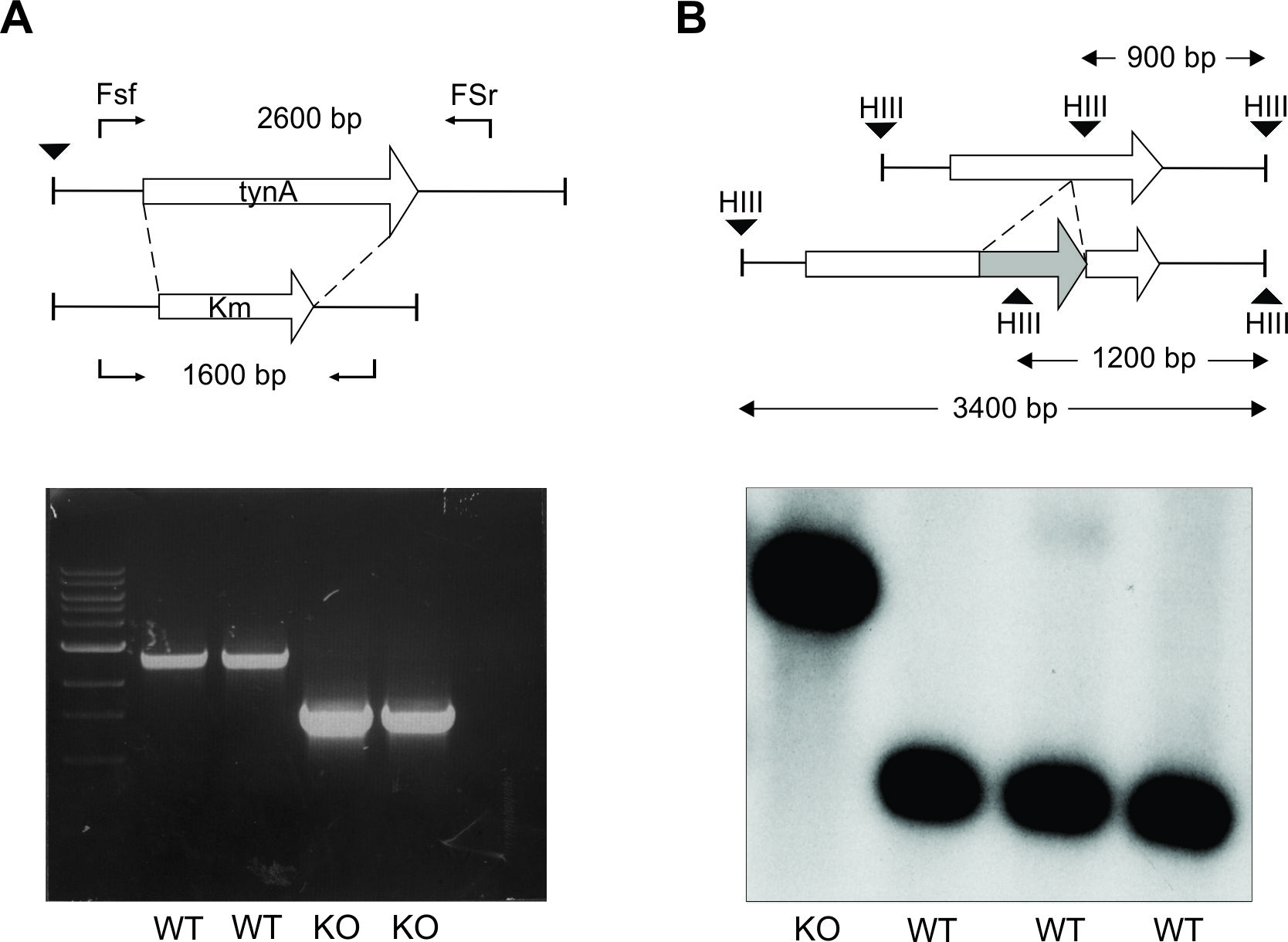


**S3 Fig. Construction of the *ΔtynA* strain.** **A**, A schematic presentation of the location of primers in relation of *tynA* and *KmR* cassette to verify *tynA* gene replacement by PCR (upper panel), and an agarose gel of a 2600 base pair (bp) band from *wt* chromosome and a 1600 bp band from Δ*tynA* chromosome amplified by PCR (lower panel). **B,** Southern hybridization visualizes the insertion in Δ*tynA*: A schematic picture of locations of HindIII (HIII) restriction sites, a 1600 bp fragment of HindIII digested Δ*tynA* chromosome and a 900 bp fragment of HindIII digested *wt* chromosome are evident on the radiograph. A second, 3400 bp hybridization fragment (common in all strains) is not shown.
