# Supplementary material for: Primary Amine Oxidase of Escherichia coli Is a Metabolic Enzyme that Can Use a Human Leukocyte Molecule as a Substrate
Source: PLoS One. 2015 Nov 10;10(11):e0142367. doi: 10.1371/journal.pone.0142367 (PMC4640556; doi:10.1371/journal.pone.0142367)
Supplement: S4 Fig — (DOCX) [file pone.0142367.s004.docx]

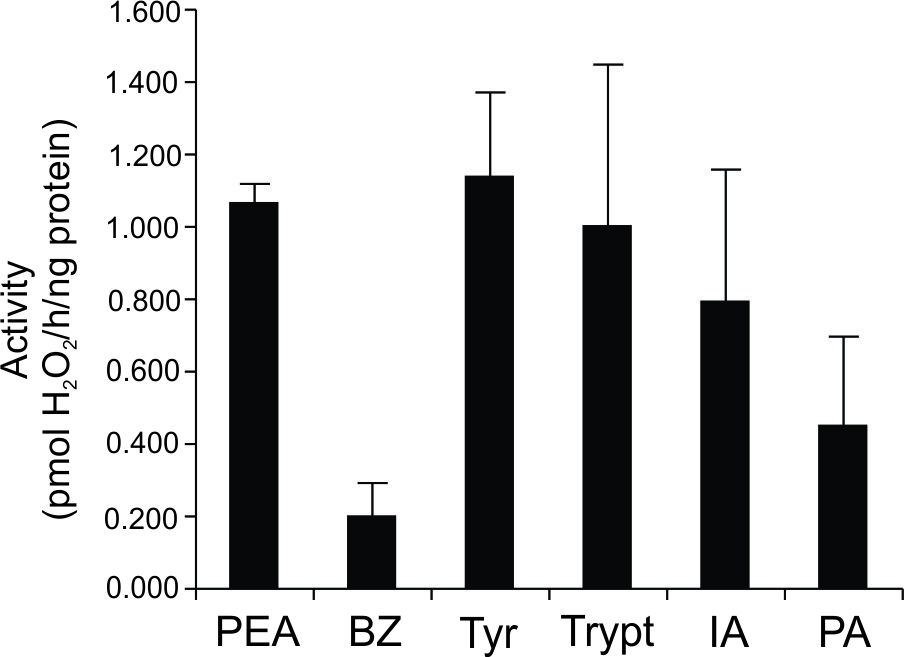


**S4 Fig.** **The activity of ECAO expressing cell lysates.** We studied the activity of ECAO on the known amine oxidase amine substrates (0.5mM): 2-phenylethylamine (PEA), benzylamine (BZ), tyramine (Tyr), tryptamine (Trypt), isoamylamine (IA) and propylamine (PA).
