# Supplementary material for: Primary Amine Oxidase of Escherichia coli Is a Metabolic Enzyme that Can Use a Human Leukocyte Molecule as a Substrate
Source: PLoS One. 2015 Nov 10;10(11):e0142367. doi: 10.1371/journal.pone.0142367 (PMC4640556; doi:10.1371/journal.pone.0142367)
Supplement: S5 Fig — (DOCX) [file pone.0142367.s005.docx]

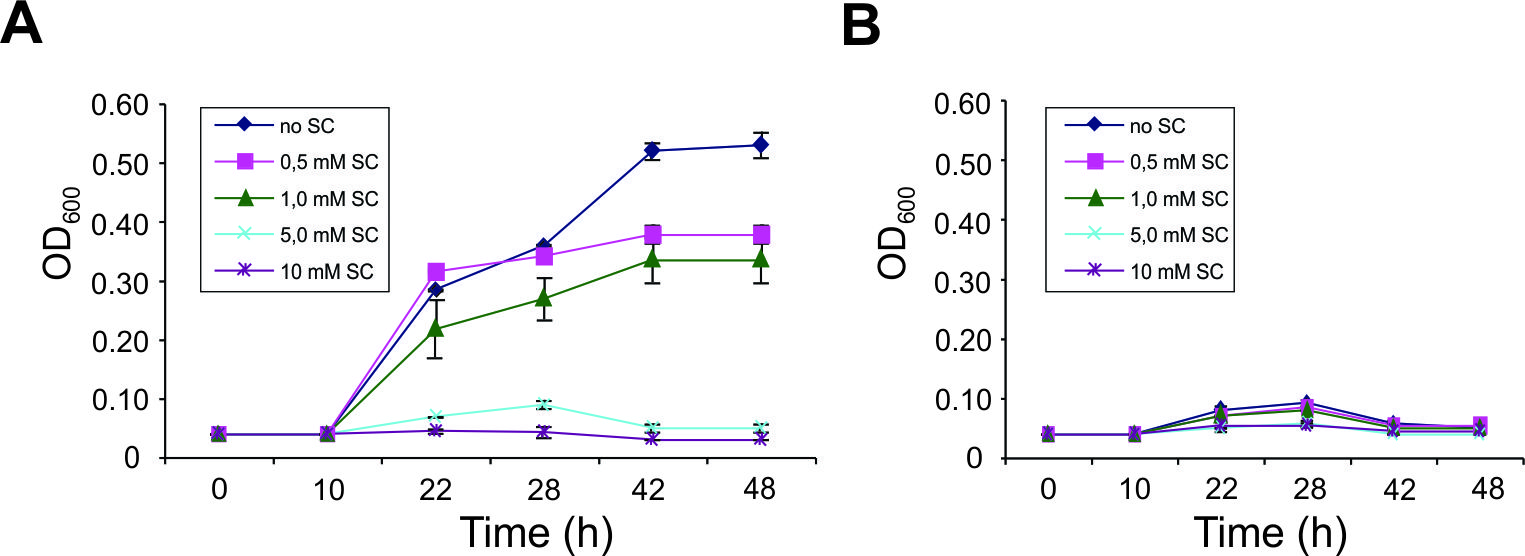


**S5 Fig. Effect of ECAO inhibition to bacterial growth.** The effect of SC on the growth of different *tynA+* (**A**) and *tynA-* (**B**) *E. coli* isolates in liquid M9-lactose-PEA medium (N=2).
