# Supplementary material for: Primary Amine Oxidase of Escherichia coli Is a Metabolic Enzyme that Can Use a Human Leukocyte Molecule as a Substrate
Source: PLoS One. 2015 Nov 10;10(11):e0142367. doi: 10.1371/journal.pone.0142367 (PMC4640556; doi:10.1371/journal.pone.0142367)
Supplement: S1 Table — (DOCX) [file pone.0142367.s006.docx]

**S1 Table.** Bacteria harboring an amine oxidase motif. * Bolded bacteria were found from soil, sea water, forest or contaminated soil.

| **Human isolates** | **Environmental isolates** |
| --- | --- |
| Acinetobacter baumannii (*) | Acetobacter pasteurianus NBRC 101655 |
| *Acinetobacter calcoaceticus* | *Acinetobacter sp.* |
| Acinetobacter nosocomialis | ***Anabaena sp. 90*** |
| Acinetobacter radioresistens | **Arthrobacter aurescens** |
| *Enterobacter aerogenes (Aerobacter aerogenes)* | ***Arthrobacter chlorophenolicus*** |
| *Enterobacter hormaechei* | **Arthrobacter globiformis** |
| Enterobacteriaceae bacterium  Escherichia coli (strain K12) + several others | **Arthrobacter sp. (strain FB24)** |
| Gordonia sputi | **Bacillus sp. 1NLA3E** |
| Klebsiella oxytoca | **Bacillus methanolicus PB1** |
| Klebsiella pneumoniae subsp. pneumoniae | **Blastococcus saxobsidens (strain DD2)** |
| Klebsiella sp. 1_ | **Catenulispora acidiphila** |
| **Klebsiella variicola** | ***Cylindrospermum stagnale*** |
| Legionella pneumophila | Enterobacter asburiae |
| Mycobacterium phlei | Enterobacter cancerogenus |
| Mycobacterium smegmatis | Enterobacter cloacae |
| ***Paenibacillus sp.*** | Enterobacter sp. Ag1 |
| *Segniliparus rugosus* | Escherichia fergusonii |
| *Shigella boydii* | Frankia sp. |
| *Yokenella regensburgei* | ***Geodermatophilus obscurus*** |
|  | Gloeocapsa sp. |
|  | **Gordonia polyisoprenivorans** |
|  | *Gordonia rhizosphera* |
|  | Hafnia alvei |
|  | **Halomonas sp. HAL1** |
|  | *Herbaspirillum seropedicae* |
|  | **Methylophaga aminisulfidivorans MP** |
|  | Microbacterium testaceum |
|  | **Modestobacter marinus** |
|  | Mycobacterium gilvum |
|  | Mycobacterium rhodesiae ?? |
|  | Mycobacterium sp. |
|  | **Mycobacterium vaccae** |
|  | ***Mycobacterium vanbaalenii (strain DSM 7251/PYR-1)*** |
|  | Nocardioidaceae bacterium |
|  | Nostoc sp. |
|  | Pseudomonas putida |
|  | **Pseudomonas sp. M47T1** |
|  | *Ralstonia solanacearum (Pseudomonas solanacearum)* |
|  | **Rhodococcus imtechensis** |
|  | *Rhodococcus opacus* |
|  | **Rhodococcus wratislaviensis** |
|  | *Saccharomonospora marina* |
|  | Shimwellia blattae |
|  | *Sphaerobacter thermophilus* |
|  | **Streptomyces bingchenggensis** |
|  | Streptomyces cattleya |
|  | Streptomyces roseosporus |
|  | ***Streptomyces sp. AA4*** |
|  | Synechocystis sp. |
|  | Trichodesmium erythraeum |
|  | Xylanimonas cellulosilytica |

*based on Swiss-Prot and TrEMBL database search on bacteria at [www.expasy.org](http://www.expasy.org).
