# Supplementary material for: Primary Amine Oxidase of Escherichia coli Is a Metabolic Enzyme that Can Use a Human Leukocyte Molecule as a Substrate
Source: PLoS One. 2015 Nov 10;10(11):e0142367. doi: 10.1371/journal.pone.0142367 (PMC4640556; doi:10.1371/journal.pone.0142367)
Supplement: S2 Table — (DOCX) [file pone.0142367.s007.docx]

**S2 Table .** ***TynA+* *E. coli* strains tested for the ECAO activity.**

| **#** | **Strain** | **Source** |
| --- | --- | --- |
| 1 | 133 | urine |
| 2 | 445 | urine |
| 3 | 462 | urine |
| 4 | 906 | urine |
| 5 | 201 | blood |
| 6 | 301 | blood |
| 7 | 360 | blood |
| 8 | 362 | blood |
| 9 | **339** | feces |
| 10 | **C-81** | feces |
| 11 | **1** | feces |
| 12 | **241** | feces |
| 13 | **DH5α** | lab |
| 14 | **8218** | feces |
| 15 | **8169** | feces |
| 16 | 8220 | feces |
| 17 | 8212 | feces |
| 18 | **1norm** | feces |
| 19 | 8213 | feces |
| 20 | **8209** | feces |
| 21 | **8204** | feces |
| 22 | **8219** | feces |
| 23 | 2 | urine |
| 24 | **32** | urine |
| 25 | 54 | urine |
| 26 | **60** | urine |
| 27 | 154 | urine |
| 28 | 155 | urine |
| 29 | 299 | urine |
| 30 | 341 | urine |
| 31 | 116 | pus |
| 32 | 150 | pus |
| 33 | 172 | pus |
| 34 | 436 | pus |
| 35 | **115** | wound |
| 36 | **120** | wound |
| 37 | 446 | pus |
| 38 | **444** | wound |
| 39 | **31** | peritoneum |

The bold ones did not show any ECAO activity. *tynA* of boxed strains were sequenced.
