# Supplementary material for: Primary Amine Oxidase of Escherichia coli Is a Metabolic Enzyme that Can Use a Human Leukocyte Molecule as a Substrate
Source: PLoS One. 2015 Nov 10;10(11):e0142367. doi: 10.1371/journal.pone.0142367 (PMC4640556; doi:10.1371/journal.pone.0142367)
Supplement: S3 Table — (DOCX) [file pone.0142367.s008.docx]

**S3 Table.** **ECAO mutations* found in *tynA+* *E. coli* strains.**

| **Strain** | **Source** | **tynA activity**** | **Mutations** |
| --- | --- | --- | --- |
| DH5α | Commercial | Inactive | K12E, G295S |
| 1 | Urine | Inactive | V283I, G364C |
| 219 | Urine | Inactive | K12E, G295S |
| 362 | Blood | Active | G295S, P311A |
| 360 | Blood | Active | D168V |
| 241 | Fecal | Inactive | none |
| C-81 | Fecal | Inactive | none |
| 445 | Urine | Active | none |

* Out of the identified mutated residues, based on the crystal structure of ECAO (PDB-code 1JRQ, Murray 2001), three were located on the surface of the molecule, the side chains facing towards the solvent (K12, V283, D168). Both mutated glycine residues were located so, that there was plenty of space to accommodate side chains for the mutations to occur (G295 and G364). Finally, P311 was located on the loop area between D1 and the rest of the molecule making a kink to the loop, but based on the structure could accommodate also a straight loop as a consequence of Pro🡪Ala mutation.

**Activity was measured using PEA as the substrate.
